# The complete chloroplast genome of *Cotyledon tomentosa* Harv.

**DOI:** 10.1080/23802359.2021.1959449

**Published:** 2021-08-16

**Authors:** Yanfeng He, Hongting Ma, Ruinan Wang, Xiangyun Gai, Pengcheng Lin, Jiaqi Zhang, Yingfang Shen

**Affiliations:** a Collage of phamacy, Qinghai Nationalities University, Xining, China; b Key Laboratory for Tibet Plateau Phytochemistry of Qinghai Province, Xining, China; c College of Ecological Environment and Resources, Qinghai Nationalities University, Xining, China

**Keywords:** Chloroplast genome, *Cotyledon tomentosa*, phylogenetic analysis

## Abstract

*Cotyledon tomentosa* Harv. is a well-known succulent plant that have important ornamental and economic value. In this study, we release and detail the complete chloroplast genome sequences of *C. tomentosa*. The whole chloroplast genome was 149,729 bp in length and comprised 131 genes, including 84 protein-coding genes, 37 tRNA genes, eight rRNA genes. The *C. tomentosa*. chloroplast genome had a GC content of 38.23%. Phylogenetic analysis based on the complete chloroplast genomes showed that *C. tomentosa* had a close relationship with *Kalanchoe tomentosa*, *Bryophyllum daigremontianum* and *Kalanchoe fedtschenkoi*.

Succulent plants, with thick and juicy leaves, increasingly attracted the attention of scientists and hobbyists alike. Most of these plants live in arid and semi-arid areas and are drought-tolerant, which is important both horticulturally and economically (Grace 2019). Coty*ledon tomentosa* Harv. (Crassulaceae) is a typical succulent plant. Its leaves have a dense layer of downy. The tips of the leaves are brown after a long time in the sun, which resembles the claws of a bear. Therefore, it is also called ‘Little Bear’ and ‘Bear Baby’. *C. tomentosa* is favored by consumers because of their diverse appearance, strong adaptability, easy reproduction, and long viewing period. It always been one of the most popular succulent species in the market.

Despite interest in *C. tomentosa* and their large ecological and economic impact, little information has been obtained to understand the mechanisms by which leaf succulence is gradually established and evolved. Information gained from complete chloroplast (cp) genome sequences and structure can explain the variation among plant species and provide valuable genomic information for the phylogenetics study (Xiong et al. 1975). But there is still only a few complete cp genome that was characterized for the species in *C. tomentosa*. This study herein releases the complete cp genome sequence of *C. tomentosa*. It would not only promote the phylogenetics study in family Crassulaceae but also provide useful genetic information for the further development of *C. tomentosa*.

The samples of *C. tomentosa* were collected in the ‘Fei-Fan gardening’, Shuyang County, Jiangsu Province, China (118°47′E, 34°06′N) and the voucher specimens (Specimen accession number: He202009, http://www.nwipb.cas.cn/znbm/qzgyswbbg/bbgjj/, Shilong Chen, herbarium@nwipb.cas.cn) are deposited in the Qinghai-Tibetan Plateau Museum of Biology, Chinese Academy of Sciences (QTPMB), Xining, China. The total DNA of *C. tomentosa* was extracted using Plant Genomic DNA Kit (DP350; TIANGEN Biotech (Beijing) Co.,Ltd.). High-throughput sequencing was performed using an Illumina NovaSeq 6000 series sequencer (PE150) by Nanjing Genepioneer Biotechnologies Inc. (Nanjing, China), and 7.73 GB of raw data was generated. The raw paired-end reads were filtered using the fastp program (Chen et al. 2018). The high-quality reads were applied to a de novo assembly performed using the program SPAdes assembler v3.10.1 (Bankevich et al. 2012). The assembled genome was annotated using CPGAVAS2 (Shi et al. 2019).

The complete cp genome (MW848817) of *C. tomentosa* was 149,729 bp in length having 38.23% of total GC content. This cp genome has a typical quadripartite structure, containing a large single-copy region (LSC) of 81,930 bp, a small single-copy region (SSC) of 16,995 bp, and 2 inverted repeat (IR) regions of 25,402 bp. A total of 131 genes are successfully annotated, including 84 protein-codon genes, 37 tRNA genes and 8 rRNA genes.

In order to reveal the phylogenetic position of *C. tomentosa* with its close relatives of Crassulaceae, a phylogenetic analysis was performed based on 33 complete chloroplast genomes of Crassulaceae. The complete chloroplast genomes were aligned by MAFFT v7.307 (Katoh and Standley 2013). The evolution history was inferred by using maximum-likelihood method based on Tamura-Nei model in MAGA7.0.26 (Kumar et al. 2016). Bootstrap value was calculated from 200 replicate analysis. Phylogenetic analysis (Figure 1) showed that *C. tomentosa* had a close relationship with *Kalanchoe tomentosa*, *Bryophyllum daigremontianum* and *Kalanchoe fedtschenkoi*.

**Figure 1.**
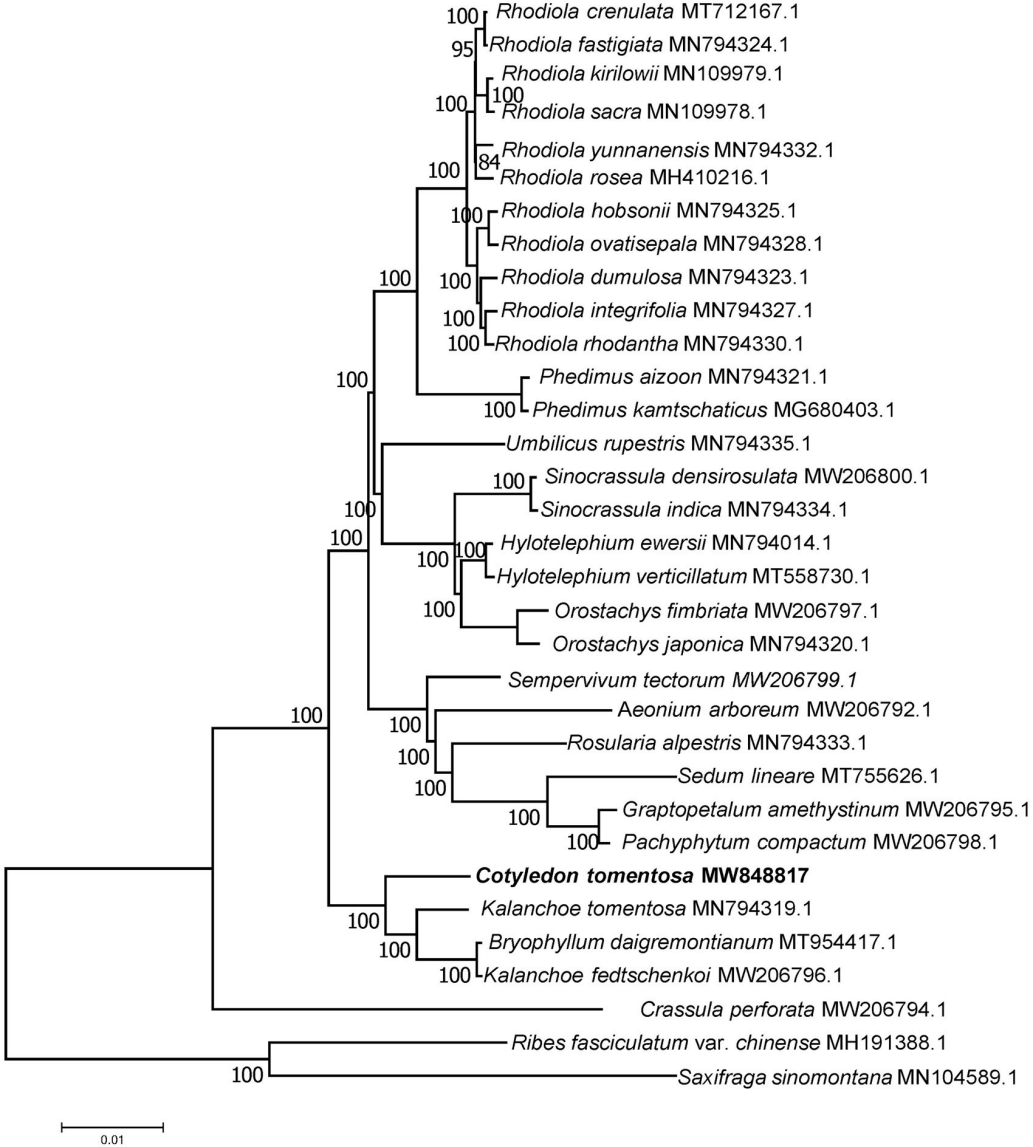
The maximum-likelihood (ML) phylogenetic tree of complete chloroplast genome sequences. Numbers above branches are bootstrap percentages based on 200 replicates.

## Data Availability

The data that support the results of this study are openly available in GeneBank (https://www.ncbi.nlm.nih.gov/genbank/) under the accession MW848817. The associated BioProject, SRA, and Bio-Sample numbers are PRJNA724941, SRR14321706, and SAMN18865650 respectively.
